# Patient Satisfaction and Oral Health-Related Quality of Life of Quadrilateral Bar versus Bilateral Linear Bar for Mandibular Implant Overdenture: Randomized Controlled Clinical Trial

**DOI:** 10.1055/s-0045-1809912

**Published:** 2025-07-08

**Authors:** Heba Wageh Abozaed

**Affiliations:** 1 Department of Prosthodontics, College of Dentistry, Prince Sattam bin Abdulaziz University, Al-Kharj, Saudi Arabia; 2 Department of Prosthodontics, Faculty of Dentistry, Mansoura University, Eldakahlia, Egypt

**Keywords:** implant, overdenture, quadrilateral bar, bilateral linear bar, satisfaction, quality of life

## Abstract

**Objective:**

This study aimed to evaluate four implant complete mandibular overdentures retained with different bar designs regarding patient satisfaction and oral health-related quality of life (OHRQoL).

**Material and Methods:**

Twenty participants were selected for this study. Each participant received four implants in the mandibular canine and first molar regions. All patients were divided into two equal groups based on their bar designs: quadrilateral (group I) and bilateral linear (group II). A visual analog scale (VAS) was used to quantify patient satisfaction, and OHRQoL was determined using the Oral Health Impact Profile (OHIP-14). Three months later, the VAS and OHIP-14 questions were evaluated.

**Statistical Analysis:**

For properly distributed data, continuous variables were shown as mean ± standard deviation. The Mann–Whitney *U* test was used to compare the two groups. The significance criterion is set at the 5% level. When *p* < 0.05, the results were deemed significant.

**Results:**

Patient satisfaction differs significantly between the two groups, as the quadrilateral bar designs enhance denture support and retention ( *p* = 0.034*) and biting or chewing food ( *p* = 0.019*). However, bilateral linear bar designs improve denture comfort ( *p* = 0.014*) and hygiene practices ( *p* = 0.007*). There were no significant variations between the two attachment designs in the remaining items of VAS and OHIP-14 questions, except that the bilateral linear bar configuration demonstrated higher scores in functional limitation ( *p* = 0.02*).

**Conclusion:**

The quadrilateral bar demonstrated greater patient satisfaction than the bilateral linear bar regarding denture stability/retention and biting or chewing food. However, the bilateral linear bars increase patient satisfaction with denture comfort and the ease of hygienic procedures. Furthermore, regarding the functional limitations of the OHIP-14 questions, the bilateral linear bar provides higher scores than the quadrilateral ones.

## Introduction

Edentulism has been described as a physical impairment affecting phonetics, function, and aesthetic perception. 1 Complete dentures have traditionally been used to rehabilitate completely edentulous individuals. However, mandibular complete dentures frequently cause issues for edentulous people. Nowadays, the implant-assisted prosthesis is a crucial component of prosthodontic treatment. 2 Implants will offer stability, retention, and aesthetic improvement, particularly in the mandible. 3

Implant overdentures come in two varieties: unsplinted (stud-type attachments) and splinted (bar attachment). 4 The interridge space, the form of the dental arch, the quantity of retention needed, the degree of implant angulation, and the cost are some variables that influence the choice of certain attachments. 5 Bars are supportive because they distribute pressures on the implants and prevent horizontal displacement forces. However, bar attachment is expensive and requires technique-sensitive manufacturing procedures. 6 Some drawbacks of the bar clip attachment include mucosal hyperplasia and hygienic issues, and clip replacement may be needed. 7

There are two primary categories of bar attachments: the rigid type, which restricts movement between the male and female components, and the resilient type, which allows for flexible movement between these parts. 8 A variety of materials can be employed for bar attachments, with plastic clips being the most favored option due to their ease of replacement chairside when retention diminishes. 9 The bar itself can be fabricated from metal, often available as prefabricated plastic patterns that are adapted to the master cast and subsequently cast in the selected alloy, or from nonmetal materials such as zirconia and polyether-ether-ketone. 8

Bar attachments are employed to splint implants, ensuring minimal complications and maximizing patient satisfaction. The stability and retention of dentures play a vital role in patient comfort and overall satisfaction for denture wearers. 10 By guiding the denture into the correct position, bar attachments enhance both retention and stability, allowing occlusal forces to be effectively distributed among the abutments. Additionally, their use improves chewing efficiency by minimizing the forward sliding of the lower denture, thereby preserving proper occlusion and reducing trauma to the underlying supporting tissues, ultimately leading to greater patient satisfaction. 11

Patient satisfaction with dentures is a critical factor in contemporary health care. Measuring satisfaction can be challenging due to its multifaceted nature and the absence of a universally accepted standard. 12 One effective method to assess satisfaction is the visual analog scale (VAS). This tool measures participants' pain levels, contentment with treatment outcomes, and overall comfort. VAS is straightforward, easy to use, reliable, and widely recognized in international literature. 13

Implant-retained or implant-supported overdentures are better than complete conventional dentures in terms of patient satisfaction regarding speech, mastication efficiency, and nutritional status. 14 Patient opinions about various elements of a specific therapy can be directly quantified through the assessment of patient satisfaction. 15 The satisfaction was influenced by several factors, the level of masticatory function, the increase in the number of dentures used before receiving an implant-supported overdenture, the type of attachment, and the number of implants used in mandibular implant-retained overdentures. 15 16

Dental research and clinical dentistry are significantly influenced by oral health-related quality of life (OHRQoL). OHRQoL refers to an individual's subjective evaluation of their oral health, including their functional and emotional well-being, expectations and satisfaction with care, and overall sense of self. This concept has various applications in both clinical and survey research. OHRQoL is an essential measure of overall health and well-being. 17

The quality of life related to dental health is evaluated using various scales. One such measure is the 14-question Oral Health Impact Profile (OHIP-14), developed by Slade et al. 18 OHRQoL can vary from individual to individual and may also change over time due to a person's evolving health status. 18

This study aimed to assess and report patient satisfaction and OHRQoL with four implant mandibular overdentures with quadrilateral and bilateral linear bars. The null hypothesis was that patient satisfaction and OHRQoL would not be different depending on the bar attachment designs (quadrilateral or bilateral linear).

## Material and Methods

### Participant Selection

**Fig. 1:**
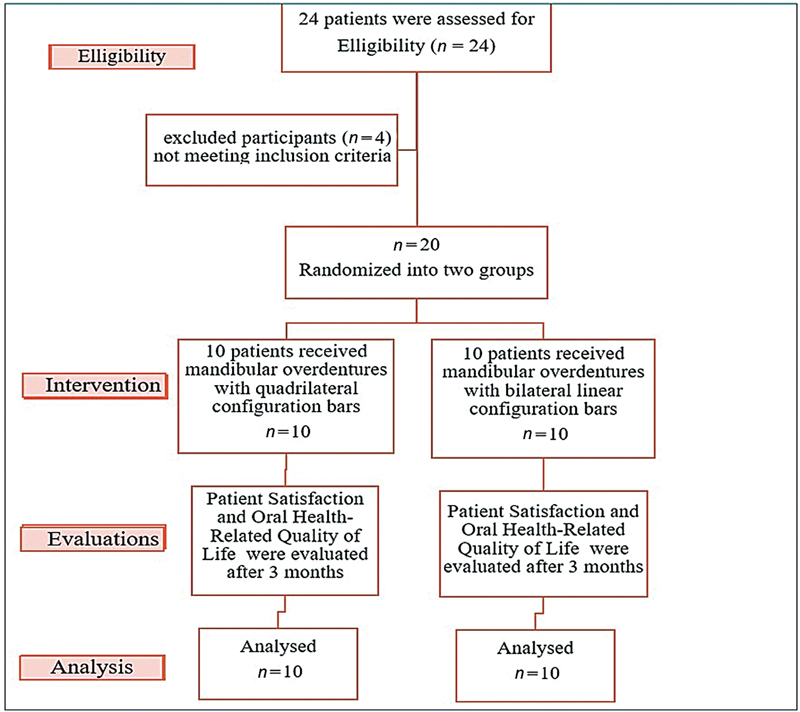
The study flowchart of participants.

Twenty complete edentate patients were chosen from the outpatient clinic of the removable prosthodontics department at the Faculty of Dentistry, Mansoura University. The participants were eligible and classified based on their characteristics (age, gender, and bone quality, Table 1 ). Based on the findings of an earlier investigation, a power of 80% was employed to compute the patient sample. 19 The G*Power software (Kiel, Germany, version 3.1.5) was used for the power analysis ( Fig. 1 ). The Ethics Committee of the Faculty of Dentistry at Mansoura University accepted the current study (No: A0101024RP), and this research was registered at ClinicalTrials.gov (NCT06673173). All the chosen participants signed written consent forms after being fully informed about all treatment plans, procedures, and necessary follow-up recalls.

**Table 1:** List of participants and their characteristics

Variable	Group	All participants (20)	Quadrilateral group (10)	Bilateral linear group (10)
Age	40–50	5	3	2
	50–60	9	3	6
	60–70	6	4	2
Gender	Male	16	9	7
	Female	4	1	3
Bone quality	D1	3	0	3
	D2	8	4	4
	D3	8	5	3
	D4	1	1	0

All patients present with healthy, firm mucosa, are completely edentulous, and show no signs of jaw cysts or residual roots. They exhibit a Class I maxillomandibular relationship, which provides sufficient restorative space and good quality of alveolar bone. 20 Individuals were excluded from the study if they had any physical, psychological, or social disabilities, as well as systemic conditions that would render minor oral surgery inadvisable, such as severe cardiovascular disease or uncontrolled type 2 diabetes. Patients with osteoporosis or a documented history of radiation treatment to the head and neck region were also excluded from the study. Moreover, individuals exhibiting specific behaviors that could jeopardize the efficacy of the treatment, such as alcoholism or the consumption of more than 10 cigarettes per day, were excluded from the study. Furthermore, patients manifesting acute or persistent symptoms of parafunctional or temporomandibular disorders were excluded from the study. 20 21

All patients received conventional complete dentures, and computer-guided surgery was performed to ensure the appropriate implant locations.

### Surgical Procedures

Using computer-guided surgery and the nonsubmerged flapless surgical method, four implants were placed (two in the canine regions and two in the first molar regions). A mandibular denture was given to each participant, and they were instructed to bite on a surgical guide to set the fixation pins. Following bone drilling, the surgical guide was taken out, implants were placed and secured with cover screws, and a panoramic radiograph was taken to assess implant locations after surgery. Following the 3-month osseointegration phase in the mandible, the dental implants were exposed, and the healing abutments were attached to the implants for 2 weeks to create the gingival collar.

### Prosthetic Procedures

An open-tray functional impression was performed for each participant. The long impression posts were fixed to the implants. To prevent movement during the removal of impressions, impression posts were splinted. Analogs were affixed to the transfer coping following the impression's removal and before the impression's pouring. Finally, a mandibular master cast was obtained.

### Bar Construction

Abutment screws were used to securely attach four plastic multiunit sleeves to the multiunit analogs on the master cast. In the quadrilateral group, Duralay resin was utilized to bond a quadrilateral three-bar assembly (multipurpose bar, Rhein OT) to the plastic abutments. Additionally, in the bilateral linear group, two bars (multipurpose bar, Rhein OT) were bonded bilaterally to the plastic abutments. The bars were cast to a cobalt-chromium alloy.

### Construction of Mandibular Overdentures

A wax occlusion rim with an acrylic base was created using the master cast. A face-bow record was taken and positioned on a semiadjustable articulator. An intermaxillary jaw relationship record was then utilized to mount the mandibular cast. Traditional flasking procedures were followed to construct the complete dentures after the teeth were set. Laboratory remounting was performed to correct any occlusal discrepancies. Additionally, intraoral occlusal contacts were evaluated to ensure that any necessary repairs could be made.

**Fig. 2:**
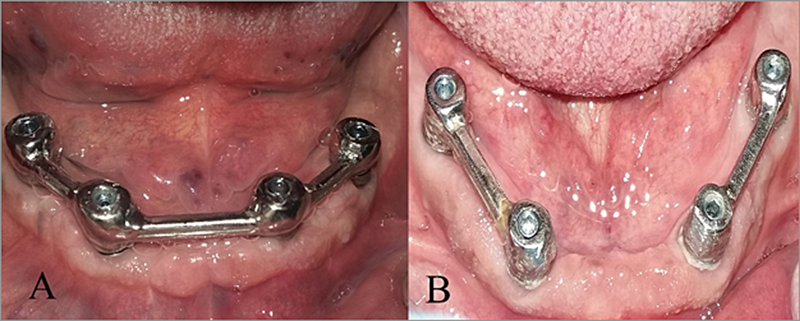
( **A** ) Intraoral quadrilateral bar attachment. ( **B** ) Intraoral linear bar attachment.

According to bar construction, patients were divided into two equal groups: group I, 10 patients received mandibular overdentures with metal quadrilateral configuration bars ( Fig. 2A ), and group II, 10 patients received mandibular overdentures with metal bilateral linear configuration bars ( Fig. 2B ). All participants received their processed dentures, which were constructed with bilateral balanced occlusion.

### Direct Functional Pickup

**Fig. 3:**
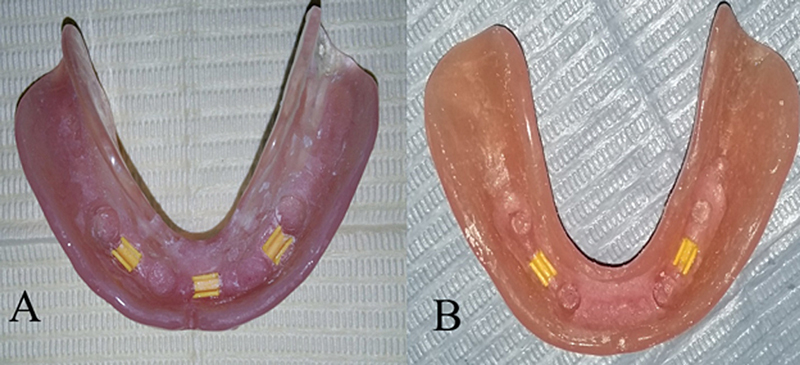
( **A** ) Pickup of clip attachment in the intaglio surface of the mandibular denture in the quadrilateral bar groups. ( **B** ) Pickup of the clip attachment in the intaglio surface of the mandibular denture in linear bar groups.

In the quadrilateral group, three yellow plastic clips were utilized—two positioned posteriorly on each side and one anteriorly (see Fig. 3A ). Additionally, two yellow plastic clips from the bilateral linear group, also with two posterior placements on each side, are illustrated in Fig. 3B . Perforations were created in the lingual flange opposite the bars to facilitate skipways for additional resin pickup material after the area beneath the bar was blocked out. While the patient maintained a centric occlusion, the clips were secured to the bars, and a direct pickup was performed using self-curing acrylic resin.

### Study Outcomes

A VAS was used to gauge participant satisfaction. The questions cover topics such as comfort level with mandibular/maxillary denture, stability, and retention of the denture, difficulty biting and chewing food, speaking difficulty, hygienic procedures, ease of handling denture, and impact on socializing. On a 100-mm scale line, participants indicated their level of satisfaction (0 representing no satisfaction at all and 100 representing complete satisfaction). The patients were given the questionnaire in Arabic. 22

The OHIP-14 ( Table 2 ): Questionnaires included seven categories: functional limits, physical aches, psychological discomforts, physical disabilities, psychological disabilities, social impairments, and handicapping. The questions were translated into Arabic. Higher scores imply reduced satisfaction, whereas lower levels indicate increasing satisfaction. The replies to the questions were never (= 1), scarcely ever (= 2), occasionally (= 3), pretty frequently (= 4), and very often (= 5). 23

**Table 2:** The questions of the Oral Health Impact Profile (OHIP-14)

Functional limitations
1. Have you had trouble pronouncing words due to dental, oral, or denture issues? (OHIP 1)
2. Have you experienced a loss of taste due to dental, oral, or denture issues? (OHIP 2)
Physical pain
3. Have you had any unpleasant oral pain or aching? (OHIP 3)
4. Have you experienced discomfort when eating due to issues with your dentures? (OHIP 4)
Psychological discomfort
5. Have you experienced self-consciousness due to teeth, mouth, or dentures? (OHIP 5)
6. Have you experienced tenseness due to dental issues or dentures? (OHIP 6)
Physical disability
7. Have you had an inadequate diet due to dental, oral, or denture issues? (OHIP 7)
8. Have you had interrupted meals due to dental or oral health issues? (OHIP 8)
Psychological disability
9. Do you have difficulty relaxing due to dental, oral, or denture issues? (OHIP 9)
10. Have you ever been embarrassed due to dental, oral, or denture problems? (OHIP 10)
Social disability
11. Have you experienced irritability due to dental or oral health issues? (OHIP 11)
12. Have oral or denture issues made it challenging to do daily tasks or jobs? (OHIP 12)
Handicap
13. Do dental, oral, or denture issues make your life less satisfying? (OHIP 13)
14. Have you been unable to function due to dental, oral, or denture problems? (OHIP 14)

Questionnaires of VAS and OHIP-14 were assessed 3 months after patients started wearing the overdentures. These 3 months were considered sufficient to enhance neuromuscular adaptation to each prosthesis. 24

## Statistical Analysis

The Statistical Package for Social Science (SPSS) application for Windows (standard version 25) was used to analyze the data. For properly distributed data, continuous variables were shown as mean ± standard deviation. The Mann–Whitney *U* test was used to compare the two groups. The significance criterion is set at the 5% level. When *p* < 0.05, the results were deemed significant.

## Results

Despite the study's short follow-up time, no patients dropped out during the evaluation phase. The results of the VAS are presented in Table 3 . The patient satisfaction results indicated no significant differences between the two groups regarding speaking with the prosthesis, denture handling ease, and effect on socializing. The quadrilateral group shows a higher statistically significant difference than the bilateral linear group regarding satisfaction with the stability and retention of the denture ( *p* = 0.034) and difficulty chewing and biting food ( *p* = 0.019). However, the bilateral linear group demonstrates a higher statistically significant difference than the quadrilateral group regarding satisfaction with denture comfort ( *p* = 0.014) and ease of hygiene procedures ( *p* = 0.007).

**Table 3:** Outcome of visual analog scale for two attachment groups

	Group I quadrilateral bar			Group II linear bar			*t* -Test	*p*
	Min-max	X ± SD	Median	Min-max	X ± SD	Median		
Denture stability and retention	80–100	95 ± 7.07	100	70–100	86 ± 9.66	85	2.12	0.034 a
Denture comfort	70–90	78 ± 7.88	80	80–100	89 ± 8.75	90	2.47	0.014 a
Speaking with a prosthesis	80–100	90 ± 8.16	90	80–100	96 ± 6.99	100	1.73	0.084
Difficulty in biting/chewing	90–100	98 ± 4.22	100	70–100	89 ± 9.94	90	2.36	0.019 a
Ease of denture handling	60–90	82 ± 10.3	85	70–100	85 ± 9.71	80	0.32	0.749
Effect on socializing	80–100	90 ± 6.66	90	90–100	95 ± 5.27	95	1.69	0.89
Ease of hygiene procedure	70–90	80 ± 8.16	80	70–100	92 ± 9.12	90	2.69	0.007 a

Abbreviations: SD, standard deviation; X, mean.

a *p* -Value is significant at the 5% level.

In the OHIP-14, Table 4 shows that there were no statistically significant differences between the two groups concerning physical pain, psychological discomfort, physical disability, psychological disability, social disability, and handicap. However, the bilateral linear group observed a higher statistically significant difference than the quadrilateral group concerning the functional limitations ( *p* = 0.02). The overall Cronbach's *α* calculated from all items is 0.774. This indicates an acceptable internal consistency among the items across both groups.

**Table 4:** Outcome of OHIP-14 for two attachment groups

	Group I	Group II	*t* -Test	*p*
	X ± SD	X ± SD		
Functional limitations	2.25 ± 0.708	1.375 ± 0.518	2.321	0.02 a
Physical pain	1.625 ± 0.518	1.25 ± 0.463	1.464	0.143
Psychological discomfort	1.875 ± 0.640	1.75 ± 0.708	0.413	0.680
Physical disability	1.5 ± 0.535	1.25 ± 0.463	1.0	0.317
Psychological disability	1.625 ± 0.518	1.375 ± 0.518	0.968	0.333
Social disability	1.25 ± 0.463	1.375 ± 0.518	0.522	0.602
Handicap	1.625 ± 0.916	1.75 ± 0.707	0.515	0.606

Abbreviations: OHIP, Oral Health Impact Profile; SD, standard deviation; X, mean.

a *p* -Value is significant at the 5% level.

## Discussion

Regardless of the attachment type, the implant overdenture enhanced patient satisfaction and OHRQoL. 25 Numerous research studies evaluate the optimal number of implants needed for a mandibular overdenture to maximize patient satisfaction. 25 Several studies propose mandibular overdentures supported by four implants to increase retention and subsequently patient satisfaction. 25 26 Although increasing the number of implants improves retention and support, it also complicates the design and raises costs and difficulties in hygiene concerns. 27 VAS is a reliable and effective tool. In this study, we used VAS as a well-established method for assessing patient satisfaction. The scale ranged from 0 to 100. 28

The degree of patient satisfaction is influenced by the retention and stability of the dentures supplied by the attachment mechanism. 29 The overdenture's retention is determined by the attachment design and material used. 14 In this study, patients reported much higher satisfaction with quadrilateral bar implant overdentures compared with bilateral linear bars regarding the stability and retention of the overdentures. This finding is consistent with previous research, which has demonstrated that splinting designs enhance retention. 30

In this study, bilateral linear bar implant overdentures had significantly superior patient satisfaction (VAS) in terms of overdenture comfort and function limitations (OHIP-14) than quadrilateral bars. That can be explained as a bilateral linear bar reducing the overcontouring of overdentures in the anterior region. That is consistent with the findings of Mahanna et al, who showed that overcontouring of the overdentures opposite the attachments restricts the tongue space. 14 As a result, patients sense the protrusion of the attachments, and the relatively large vertical dimension of the anterior bar may trigger the periosteal mechanoreceptors near the dental implant, possibly accountable for the sensation that the prosthesis was not a part of them. 31

Improved denture retention and stability allow for the restoration of oral function like mastication. 32 In this study, patients reported significantly higher satisfaction with quadrilateral bar implant overdentures in terms of biting and chewing difficulties compared with those with bilateral linear bars. This improvement is attributed to the increased retention provided by the quadrilateral bar distribution. These findings align with those of Elsyad et al, who noted that the enhanced retention and stability of overdentures supported by bar anchors led to greater muscular activation. 31

Bilateral linear configuration overdentures have shown significantly higher satisfaction regarding oral hygiene compared with quadrilateral bar overdentures. This increased satisfaction may be attributed to greater mucosal coverage. Boven et al noted that the bar system tends to harbor more plaque biofilms, which complicates cleaning around the implant for the patient. 33 Furthermore, the areas around the bar and abutments provide a favorable region for bacteria and plaque accumulation. 34

The study found that there was no significant difference in most items of OHRQoL between the groups. The short evaluation duration (3 months) could be the reason for the small variance in patient OHRQoL variations between attachments, as it is insufficient to cause complications of the retentive components.

The limitations of this study include the inability to blind participants to the treatment, which introduces the possibility of bias. The study did not consider the influence of different types of dentitions, such as maxillary complete dentures, fixed dentures, partial dentures, implant overdentures, or natural teeth. Moreover, most participants had low educational levels. Although all dentures were worn for 3 months, the impact of wear time on patient satisfaction was not evaluated. Furthermore, the study did not analyze the magnitude of significant differences or effect sizes. Future studies will require longer observation periods, greater sample sizes, and further clinical aspects regarding the topic of the current study.

## Conclusion

Within the constraints of this study, the rehabilitation of edentulous mandibles using two designs of implant overdenture bars (quadrilateral and bilateral linear) demonstrated acceptable levels of patient satisfaction and OHRQoL. The quadrilateral bar was associated with higher patient satisfaction in terms of denture stability and retention, as well as the ability to bite and chew food. In contrast, the bilateral linear bar enhanced comfort and facilitated hygienic procedures in addition to the functional limitations of OHRQoL. Conversely, no significant differences were observed in other aspects of OHRQoL between the two groups.

## Recommendations

More long-term studies of variant evaluation methods are thus required to validate the results of this study.

## References

[1] Hsu Y J Lin J R Hsu J F Patient satisfaction, clinical outcomes and oral health-related quality of life after treatment with traditional and modified protocols for complete dentures J Dent Sci 2021 16 01 236 240 33384803 10.1016/j.jds.2020.05.024 PMC7770313

[2] Bakker M H Vissink A Meijer H JA Raghoebar G M Visser A Mandibular implant-supported overdentures in (frail) elderly: a prospective study with 20-year follow-up Clin Implant Dent Relat Res 2019 21 04 586 592 30993810 10.1111/cid.12772 PMC6767521

[3] Doornewaard R Glibert M Matthys C Vervaeke S Bronkhorst E de Bruyn H Improvement of quality of life with implant-supported mandibular overdentures and the effect of implant type and surgical procedure on bone and soft tissue stability: a three-year prospective split-mouth trial J Clin Med 2019 8 06 773 31159202 10.3390/jcm8060773 PMC6617188

[4] Salehi R Shayegh S S Johnston W M Hakimaneh S MR Effects of interimplant distance and cyclic dislodgement on retention of LOCATOR and ball attachments: an in vitro study J Prosthet Dent 2019 122 06 550 556 31027962 10.1016/j.prosdent.2018.12.023

[5] Sutariya P V Shah H M Patel S D Upadhyay H H Pathan M R Shah R P Mandibular implant-supported overdenture: a systematic review and meta-analysis for optimum selection of attachment system J Indian Prosthodont Soc 2021 21 04 319 327 34810359 10.4103/jips.jips_158_21 PMC8617439

[6] Ciftci G Somay S D Ozcan I Ozcelik T B Yilmaz B Prosthetic complications with mandibular bar-retained implant overdentures having distal attachments and metal frameworks: a 2- to 12-year retrospective analysis J Prosthet Dent 2023 130 04 573 580 34998584 10.1016/j.prosdent.2021.11.016

[7] Chae S K Cho W T Choi J W Comparison of retentive force and wear pattern of Locator® and ADD-TOC attachments combined with CAD-CAM milled bar J Adv Prosthodont 2022 14 01 12 21 35284053 10.4047/jap.2022.14.1.12 PMC8891684

[8] Fromentin O Lassauzay C Abi Nader S Feine J de Albuquerque Junior R F Testing the retention of attachments for implant overdentures - validation of an original force measurement system J Oral Rehabil 2010 37 01 54 62 19912482 10.1111/j.1365-2842.2009.02020.x

[9] Emera R MK Altonbary G Y Retention force of zirconia bar retained implant overdenture: clinical comparative study between PEEK and plastic clips Int Dent Res 2019 9 92 98

[10] Nassar H I Abdelaziz M S Retention of bar clip attachment for mandibular implant overdenture BMC Oral Health 2022 22 01 227 35681163 10.1186/s12903-022-02262-7 PMC9178882

[11] Botega D M Mesquita M F Henriques G EP Vaz L G Retention force and fatigue strength of overdenture attachment systems J Oral Rehabil 2004 31 09 884 889 15369470 10.1111/j.1365-2842.2004.01308.x

[12] Brokelman R BG Haverkamp D van Loon C Hol A van Kampen A Veth R The validation of the visual analogue scale for patient satisfaction after total hip arthroplasty Eur Orthop Traumatol 2012 3 02 101 105 22798966 10.1007/s12570-012-0100-3 PMC3389603

[13] Tosun B Uysal N Examination of oral health quality of life and patient satisfaction in removable denture wearers with OHIP-14 scale and visual analog scale: a cross-sectional study BMC Oral Health 2024 24 01 1353 39511586 10.1186/s12903-024-05124-6 PMC11545866

[14] Mahanna F F Elsyad M A Mourad S I Abozaed H W Satisfaction and oral health-related quality of life of different attachments used for implant-retained overdentures in subjects with resorbed mandibles: a crossover trial Int J Oral Maxillofac Implants 2020 35 02 423 431 32142580 10.11607/jomi.7869

[15] Yunus N Saub R Taiyeb Ali T B Salleh N M Baig M R Patient-based and clinical outcomes of implant telescopic attachment-retained mandibular overdentures: a 1-year longitudinal prospective study Int J Oral Maxillofac Implants 2014 29 05 1149 1156 25216142 10.11607/jomi.3328

[16] Kucukkurt S Tùkel H C Does the number of implants or the type of attachment affect patient satisfaction with implant-retained mandibular overdentures? J Osseointeg 2020 12 154 160

[17] Chai H H Gao S S Chen K J Lo E CM Duangthip D Chu C H Tools evaluating child oral health–related quality of life Int Dent J 2024 74 01 15 24 37482502 10.1016/j.identj.2023.07.004 PMC10829350

[18] Slade G D Derivation and validation of a short-form oral health impact profile Community Dent Oral Epidemiol 1997 25 04 284 290 9332805 10.1111/j.1600-0528.1997.tb00941.x

[19] Elsyad M A Alokda M M Gebreel A A Hammouda N I Habib A A Effect of two designs of implant-supported overdentures on peri-implant and posterior mandibular bone resorptions: a 5-year prospective radiographic study Clin Oral Implants Res 2017 28 10 e184 e192 27637737 10.1111/clr.12984

[20] Mourad K E Emera R MK Habib A Effect of different implant positions for two implant-retained mandibular overdenture: a retrospective 5-years radiographic evaluation of the circumferential peri-implant bone loss and posterior ridge resorptive changes BMC Oral Health 2024 24 01 1161 39350107 10.1186/s12903-024-04871-w PMC11443777

[21] de Souza R F Bedos C Esfandiari S Single-implant overdentures retained by the Novaloc attachment system: study protocol for a mixed-methods randomized cross-over trial Trials 2018 19 01 243 29685161 10.1186/s13063-018-2606-7 PMC5913792

[22] Elsyad M A Patient satisfaction and prosthetic aspects with mini-implants retained mandibular overdentures. A 5-year prospective study Clin Oral Implants Res 2016 27 07 926 933 26129836 10.1111/clr.12660

[23] Elsyad M A Mostafa A Z Effect of telescopic distal extension removable partial dentures on oral health related quality of life and maximum bite force: a preliminary cross over study J Esthet Restor Dent 2018 30 01 14 21 28891594 10.1111/jerd.12325

[24] Elsyad M A Khairallah A S Chewing efficiency and maximum bite force with different attachment systems of implant overdentures: a crossover study Clin Oral Implants Res 2017 28 06 677 682 27118683 10.1111/clr.12861

[25] Kim H Y Lee J Y Shin S W Bryant S R Attachment systems for mandibular implant overdentures: a systematic review J Adv Prosthodont 2012 4 04 197 203 23236571 10.4047/jap.2012.4.4.197 PMC3517957

[26] Elsyad M A Hegazy S AF Hammouda N I Al-Tonbary G Y Habib A A Chewing efficiency and electromyographic activity of masseter muscle with three designs of implant-supported mandibular overdentures. A cross-over study Clin Oral Implants Res 2014 25 06 742 748 23445173 10.1111/clr.12137

[27] Singla N Evaluation of patient satisfaction in implant-supported dentures retained with ball and bar attachment through verbal rating system Int J Exp Dent Sci 2011 6 1 5

[28] Flytström I Stenberg B Svensson Å Bergbrant I M Patients' visual analogue scale: a useful method for assessing psoriasis severity Acta Derm Venereol 2012 92 04 347 348 22101827 10.2340/00015555-1237

[29] Helmy M Elkhadem A Validation of maintenance requirements and patient satisfaction of classical implant locators versus customized attachments in mandibular implant-retained overdentures Egypt J Oral& Maxillofac Sur 2023 14 66 75

[30] Takahashi T Gonda T Tomita A Maeda Y Effect of attachment type on implant strain in maxillary implant overdentures: comparison of ball, locator, and magnet attachments. part 2: palateless dentures Int J Oral Maxillofac Implants 2018 33 02 357 364 29534124 10.11607/jomi.6157

[31] Elsyad M A Khairallah A S Shawky A F Changes in the edentulous maxilla with ball and telescopic attachments of implant-retained mandibular overdentures: a 4-year retrospective study Quintessence Int 2013 44 07 487 495 23616978 10.3290/j.qi.a29612

[32] Limpuangthip N Somkotra T Arksornnukit M Modified retention and stability criteria for complete denture wearers: a risk assessment tool for impaired masticatory ability and oral health-related quality of life J Prosthet Dent 2018 120 01 43 49 29195820 10.1016/j.prosdent.2017.09.010

[33] Boven G C Meijer H JA Vissink A Raghoebar G M Maxillary implant overdentures retained by use of bars or locator attachments: 1-year findings from a randomized controlled trial J Prosthodont Res 2020 64 01 26 33 31201036 10.1016/j.jpor.2019.04.013

[34] Lachmann S Kimmerle-Müller E Gehring K A comparison of implant-supported, bar- or ball-retained mandibular overdentures: a retrospective clinical, microbiologic, and immunologic study of 10 edentulous patients attending a recall visit Int J Prosthodont 2007 20 01 37 42 17319360

